# Impact of open femoral endarterectomy on treating multilevel iliac and common femoral artery occlusive disease

**DOI:** 10.3389/fsurg.2025.1445846

**Published:** 2025-01-21

**Authors:** Suehyun Park, Taewan Ku, Deokbi Hwang, Woo-Sung Yun, Seung Huh, Hyung-Kee Kim

**Affiliations:** ^1^ Division of Vascular and Endovascular Surgery, Department of Surgery, Kyungpook National University Hospital, Daegu, Republic of Korea; ^2^ Division of Vascular and Endovascular Surgery, Department of Surgery, Kyungpook National University Chilgok Hospital, School of Medicine, Kyungpook National University, Daegu, Republic of Korea

**Keywords:** peripheral arterial disease, ischemia, treatment outcome, endarterectomy, stent, common femoral artery

## Abstract

**Purpose:**

This study aimed to evaluate the impact of femoral endarterectomy (FE) on treating multilevel iliac and common femoral artery occlusive disease.

**Materials and methods:**

From January 2013 to December 2022, 106 limbs in 103 patients with multilevel arterial occlusive disease underwent open FE and iliac angioplasty (FEIA) with or without infrainguinal revascularization. The primary outcome assessment was the changes in the TransAtlantic Inter-Society Consensus (TASC) II classification during the operation; the secondary outcomes included the primary patency (PP) and secondary patency (SP) of FEIA. The risk factors for PP loss were evaluated.

**Results:**

Of the 103 patients, 91 were male. A total of 56 limbs were treated for chronic limb-threatening ischemia, and 61 limbs underwent infrainguinal revascularization. Preoperatively, aortoiliac occlusive disease (AIOD) was classified as TASC II C in 65 (61%) limbs and D in 41 limbs. During the operation, 19 limbs received additional thrombectomy for subacute or chronic thrombus components. Overall, FE and additional thrombectomy reduced the TASC II classification of AIOD from complex lesions (TASC II C/D) to simple lesions (B or lesser) in 101 (95%) of 106 limbs. Three early mortalities (2.8%, two from acute myocardial infarctions and one from pneumonia) were recorded. The PP and SP of FEIA were 89% and 96% at 1 year, 80% and 94% at 3 years, and 77% and 94% at 5 years, respectively. The severity of iliac and common femoral artery disease was not associated with PP loss of FEIA.

**Conclusions:**

Despite the challenging nature of initially classified TASC II C/D lesions, our findings highlight the effectiveness of FE in reducing TASC II classification and the durable patency achieved with FEIA. Hybrid FEIA could be a viable primary treatment option, particularly for lesions featuring severe iliac and common femoral artery disease.

## Introduction

The classification system for the severity of aortoiliac occlusive disease (AIOD) categorizes lesions involving the common femoral artery (CFA) as TransAtlantic Inter-Society Consensus (TASC) II C/D lesions due to the complexity of endovascular treatment (EVT) and lower patency rates compared with TASC II A/B lesions (1). In addition, the recently published Global Limb Anatomic Staging System (GLASS) categorizes lesions with over 50% stenosis in the CFA as more complex type B lesions (2). While advancements in endovascular techniques and equipment have led to improved outcomes of EVT for complex aortoiliac lesions, including those involving the CFA, the rates of technical failure and restenosis during follow-up have been found to be higher than those for simpler lesions (TASC II A/B lesions) (3–6).

Another option for treating these complex lesions involving the CFA is a hybrid approach, combining open femoral endarterectomy (FE) and endovascular iliac angioplasty (FEIA). The primary advantages of this approach include reduced invasiveness compared to traditional open surgery and higher technical success rates compared to EVT alone. In addition, it may contribute to increased patency rates, given that the surgical outcomes for atherosclerotic CFA disease tend to be superior to those of EVT in cases of isolated CFA disease (7, 8). Furthermore, with the widespread adoption of hybrid operating rooms, an increasing number of hybrid procedures in peripheral arterial disease, including FEIA, have been performed with favorable outcomes (9, 10).

While there is abundant literature reporting on patency, documentation regarding changes in TASC classification during operations is very rare (11). This gap underscores the need for more detailed examination and reporting on classification shifts during operations, which could provide deeper insights into treatment effectiveness and decision-making.

In this study, we aim to evaluate the efficacy of FE in multilevel iliac and CFA occlusive disease with respect to changes in TASC II classification during the operation. In addition, we analyze the primary patency (PP) and secondary patency (SP) of FEIA and the risk factors for PP loss.

## Materials and methods

### Data source and variables

This study was approved by the local institutional review board (IRB No. KNUCH 2022-04-013) and was exempt from the requirement of informed consent. It included 106 limbs from 103 patients with multilevel arterial occlusive disease who underwent hybrid FEIA, with or without infrainguinal revascularization, between January 2013 and December 2022. To explore the additional role of FE, we also included patients who received thrombectomy for subacute or chronic thrombus components detected intraoperatively. However, patients with acute limb ischemia were excluded.

After reviewing the patients’ medical charts and images, we collected the following information: patient characteristics, previous history of ipsilateral revascularization, imaging findings, surgical details, and follow-up results. Aortoiliac lesion characteristics were primarily evaluated using computed tomography (CT) scans, while preoperative duplex ultrasonography (DUS) was utilized for patients with renal insufficiency. In addition, initial intraoperative angiography was also used for evaluation. Based on the presence of occlusion in the iliac artery and CFA and the lesion length, we classified the lesions according to the TASC II classification (1). In addition, we assessed the patency status of outflow vessels, such as the superficial and deep femoral arteries, and classified the severity of femoropopliteal disease according to the TASC II classification of femoropopliteal lesions (1).

Perioperative complications were categorized as local/non-vascular, local/vascular, or systemic/remote and were graded according to the recommended standards (12, 13). During the follow-up period, we recorded the patency of the FEIA, any reinterventions of the index limb, above-the-ankle amputations, and survival data.

### Operative details and follow-up protocol

The FEIA procedure adhered to standard operative techniques. In cases with subacute or chronic thrombus detected intraoperatively, retrograde surgical thrombectomy via femoral arteriotomy using a Fogarty catheter was added. Subsequently, FE was performed, followed by patch angioplasty to close the arteriotomy site. Profundaplasty was performed at the surgeon's discretion for proximal deep femoral artery (DFA) disease. In patients without infrainguinal bypass or those undergoing EVT for infrainguinal disease, patch angioplasty was preferred for closing the endarterectomy site; however, in select patients requiring infrainguinal bypass, the endarterectomy site was used as the inflow of the bypass without a patch.

For the iliac angioplasty, retrograde wire passage and subsequent angiography were conducted, followed by iliac angioplasty in stenotic lesions. In occluded lesions, a contralateral CFA puncture using an up-and-over technique or a brachial approach with cutdown was conducted for failed retrograde wire passages. In most iliac lesions, primary stenting was performed.

The postoperative follow-up protocol included: (1) ankle-brachial index (ABI) before discharge; (2) DUS and ABI at 1 month; (3) clinical follow-up at 3 months; (4) ABI at 6 months; (5) DUS and ABI at 1 year; and (6) DUS and ABI annually. If symptoms worsened or the ABI value decreased by more than 0.15, additional DUS or CT was performed. Patients who returned with symptoms before their scheduled follow-up were evaluated using the same protocol, with other procedures for correction as required. After 1 year postoperatively, the dual antiplatelet therapy was typically changed to single antiplatelet therapy.

### Outcomes of interest and definitions

Recognizing that, according to TASC II guidelines, every patient in our series had CFA disease, lesions were automatically classified into either C or D status. In this study, AIOD severity was determined by examining a unilateral lesion without considering the quality of AIOD on the opposite side. In alignment with TASC II guidelines, TASC D includes diffuse multiple stenoses involving the unilateral common iliac artery (CIA), external iliac artery (EIA), and CFA, or unilateral occlusion of both the CIA and EIA. Lesions in our series categorized as TASC C comprised the remaining lesions not categorized as TASC D. During the operations, the TASC classification of the lesions was reassessed after FE; additionally, the final TASC classification before iliac stenting was further assessed through angiography, including patients with additional thrombectomy. We compared the changes in TASC classification as follows: preoperative, after FE, and final results before iliac stenting.

The efficacy outcome was the PP and SP of the FEIA. Risk factors influencing PP were further assessed according to patient and lesion characteristics and operative variables. The safety outcome was the perioperative morbidity and mortality rates within 30 days post-procedure, classified as grade 1, 2, or 3 according to the recommended reporting standard for lower-extremity ischemia (12, 13).

PP was defined as uninterrupted FEIA site patency without occlusion or reintervention, including both endovascular and surgical procedures. SP referred to FEIA site patency following occlusion after a successful endovascular or surgical procedure. Major amputations were those at or above the ankle.

### Statistical analysis

After the normality test, the student’s *t*-test or Mann–Whitney *U* test was used to assess the continuous variables, while the Chi-square test (for adequate sample sizes) or Fisher exact test (for smaller sample sizes) was used to analyze the categorical variables. The PP and SP rates were evaluated using Kaplan–Meier plots. The statistical significance of the differences between the survival curves was ascertained using the log-rank test. Furthermore, independent risk factors for PP were identified by Cox regression analysis. All statistical results were analyzed using IBM SPSS (v. 23.0; IBM Corporation), and a *P*-value <0.05 was considered significant.

## Results

### Patient and lesion characteristics

A total of 106 FEIAs were performed on 103 consecutive patients, whose mean age was 71.8 ± 8.8 years (range: 43–90 years). Out of them, 91 patients were male. A total of 56 (52.8%) limbs had chronic limb-threatening ischemia (CLTI) (with rest pain in 22 limbs, minor tissue loss in 30 limbs, and major tissue loss in 4 limbs), and 50 limbs presented with disabling claudication. Table 1 provides an overview of the patients’ characteristics. Patients with CLTI exhibited higher prevalence of the female sex, were of older age, and had renal insufficiency compared to those with claudication, and also had lower ABI values. In total, 30 ipsilateral revascularization procedures were performed on 22 patients, involving iliac angioplasty (*n* = 12), infrainguinal EVT (*n* = 10), infrainguinal bypass (n=6), and femorofemoral bypass (*n* = 2). None of the included patients had previously undergone FE.

**Table 1:** Patients’ characteristics.

	Total (*N* = 106)	Claudication (*N* = 50)	CLTI (*N* = 56)	*P*-value
Male	91 (86%)	49 (98%)	42 (75%)	0.001
Age	71.8 ± 8.8	69.5 ± 7.6	73.8 ± 9.4	0.012
Hypertension	82 (77%)	37 (74%)	45 (80%)	0.435
Diabetes mellitus	48 (45%)	22 (44%)	26 (46%)	0.802
Coronary artery disease	38 (36%)	17 (34%)	21 (38%)	0.708
Congestive heart failure	12 (11%)	4 (8%)	8 (14%)	0.308
Cerebrovascular disease	30 (28%)	14 (28%)	16 (29%)	0.948
Chronic obstructive lung disease	16 (15%)	8 (16%)	8 (14%)	0.806
Renal insufficiency^a^	40 (38%)	14 (28%)	26 (46%)	0.005
Dialysis	8 (8%)	2 (4%)	6 (11%)	0.277
eGFR	70.1 ± 34.5	75.0 ± 30.9	65.7 ± 37.1	0.168
Dyslipidemia	62 (59%)	32 (64%)	30 (54%)	0.277
ASA classification				0.695
1	3	1	2	
2	43	22	21	
3	59	27	32	
4	1	0	1	
Previous revascularization	30 (28%)	9 (18%)^b^	21 (38%)^c^	0.105
Iliac EVT		4	8	
Femorofemoral bypass		1	1	
Infrainguinal bypass		3	3	
Infrainguinal EVT		1	9	
Preoperative ABI	0.54 ± 0.29	0.64 ± 0.23	0.44 ± 0.32	0.001

CLTI, chronic limb-threatening ischemia; eGFR, estimated glomerular filtration rate; ABI, ankle-brachial index; ASA, American Society of Anesthesiologists; EVT, endovascular therapy.

^a^ eGFR <60 ml/min/1.73 m^2.^

^b^ Two patients received both of the procedures listed.

^c^ Six patients received both of the procedures listed.

**Figure 1:**
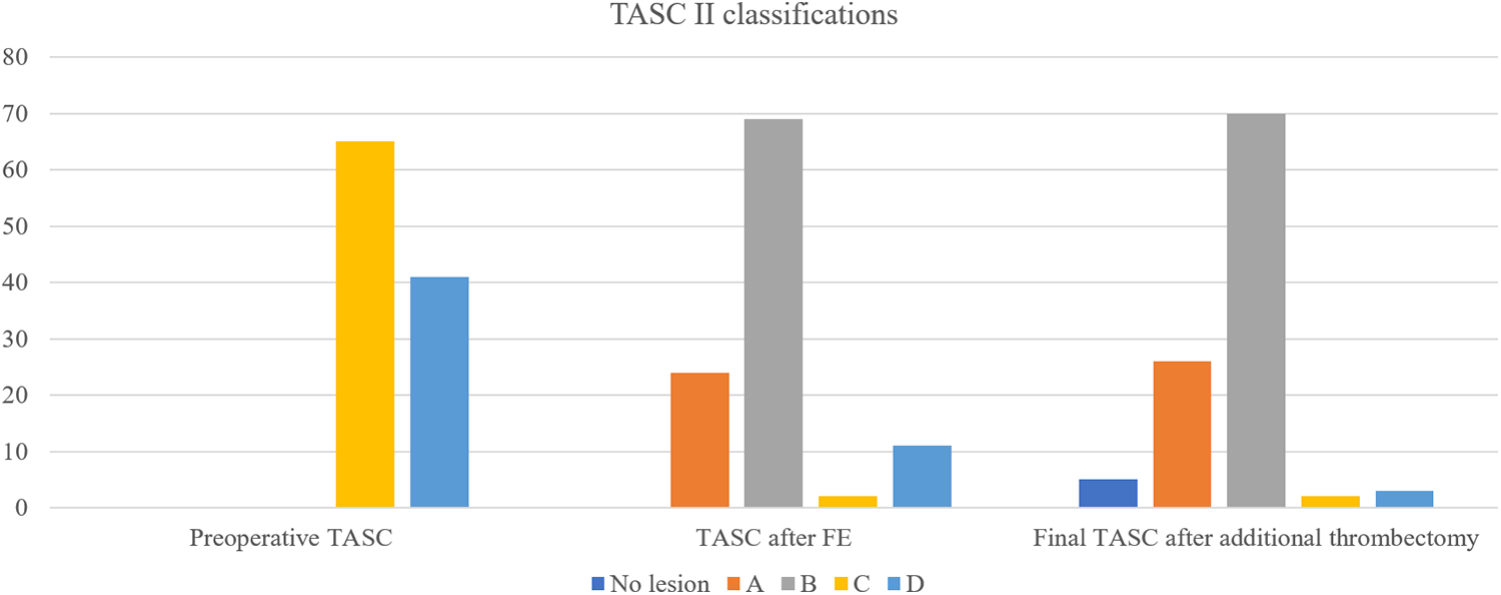
Changes in TASC II classifications before and during the hybrid procedure.

Table 2 details the lesion characteristics. Regarding iliac lesions, 27 (26%) limbs displayed total occlusion, while the remaining 79 exhibited stenosis. Iliac occlusion was located in the CIA in 6 limbs, EIA in 9 limbs, and both CIA and EIA in 12 limbs. CFA occlusion was present in 35 (33%) extremities, and femoropopliteal occlusion was identified in 55 (52%) extremities. Based on the TASC II classification, AIOD was classified as C in 65 (61%) limbs and D in 41 limbs (Figure 1).

**Table 2:** Lesion characteristics.

	Total (*N* = 106)	Claudication (*N* = 50)	CLTI (*N* = 56)	*P*-value
Aortoiliac TASC II classification				0.792
C	65 (61%)	30 (60%)	35 (62.5%)	
D	41 (39%)	20 (40%)	21 (37.5%)	
Iliac artery occlusion	27 (25%)	9 (18%)	18 (32%)	0.095
CIA only	6	2	4	
EIA only	9	4	5	
Both CIA and EIA	12	3	9	
IIA occlusion	46 (44%)	23 (46%)	23 (42%)	0.666
CFA occlusion	35 (33%)	15 (30%)	20 (36%)	0.532
DFA occlusion	8 (8%)	2 (4%)	6 (11%)	0.277
FP occlusion	55 (52%)	18 (36%)	37 (66%)	0.002
FP TASC II classification				0.020
No lesion	18	13	5	
A	8	6	2	
B	12	7	5	
C	31	10	21	
D	37	14	23	

CLTI, chronic limb-threatening ischemia; TASC, TransAtlantic Inter-Society Consensus; CIA, common iliac artery; EIA, external iliac artery; IIA, internal iliac artery; CFA, common femoral artery; DFA, deep femoral artery; FP femoropopliteal.

### Operative characteristics

Table 3 summarizes the operative characteristics. Regarding iliac angioplasty, stent placement was performed in 88 (83%) limbs, balloon angioplasty alone in 9 limbs, and stent graft placement in 4 limbs. In addition, 19 limbs underwent iliac artery thrombectomy. Notably, in five of these limbs, iliac thrombus resolution without definite stenosis led to the decision not to perform iliac angioplasty.

**Table 3:** Operation details.

	Total (*N* = 106)	Claudication (*N* = 50)	CLTI (*N* = 56)	*P*-value
Iliac angioplasty	101	49 (98%)	52 (93%)	0.367^a^
Stent deployment	88	43 (86%)	45 (80%)	0.440
Stent graft deployment	4	3 (6%)	1 (2%)	0.341^a^
Balloon angioplasty alone	9	3 (6%)	6 (11%)	0.495^a^
Iliac thrombectomy alone	5	1 (2%)	4 (7%)	0.367^a^
FE and angioplasty				
Patch use	101	47 (94%)	54 (96%)	0.665^a^
Profundaplasty	30	9 (18%)	21 (38%)	0.026
Infrainguinal revascularization	61	23 (46%)	38 (68%)	0.023
Infrainguinal EVT	35	13 (26%)	22 (39%)	0.147
Infrainguinal bypass	23	9 (18%)	14 (25%)	0.383
Thrombectomy	5	2 (4%)	3 (5%)	1.000^a^
Kinds of iliac stent and stent graft				
SES	78	39 (78%)	39 (70%)	0.330
BES	12	4 (8%)	8 (14%)	0.308
Both of BES and SES	2	0	2 (4%)	1.000^a^
BESG	4	3 (6%)	1 (2%)	0.341^a^
Length of stent (median, mm)		80.0	60.0	0.083
Postoperative ABI	0.88 ± 0.20	0.90 ± 0.18	0.85 ± 0.23	0.243

CLTI, chronic limb-threatening ischemia; FE, femoral endarterectomy; EVT, endovascular therapy; SES, self-expanding stent; BES, balloon-expandable stent; BESG, balloon-expandable stent graft; ABI, ankle-brachial index.

^a^ Fisher’s exact test.

Regarding the surgical details of the CFA lesions, 101 limbs received FE with patch angioplasty. However, five limbs underwent only FE, with the endarterectomy site utilized for infrainguinal bypass inflow. The most common material for patch angioplasty was a bovine pericardial patch, followed by a polytetrafluoroethylene (PTFE) patch. In addition, 30 limbs underwent profundaplasty.

After FE, the TASC II classification of AIOD changed to A, B, C, and D in 24, 69, 2, and 11 limbs, respectively. After additional thrombectomy, the final TASC II classifications before iliac intervention were as follows: no iliac lesions in 5 patients, A in 26 limbs, B in 70 limbs, C in 2 limbs, and D in 3 limbs (Figure 1). Overall, FE reduced the TASC II classification of AIOD from complex lesions (TASC II C/D) to simple lesions (B or lesser) in 101 (95%) of 106 limbs.

Infrainguinal revascularization was performed in 61 (57.5%) limbs. Infrainguinal EVT was applied to 35 limbs, followed by infrainguinal bypass in 23 limbs and surgical thrombectomy alone in 3 limbs. Patients with CLTI were more likely to undergo infrainguinal revascularization (68% vs. 46%, *P* = 0.023) and profundaplasty (38% vs. 18%, *P* = 0.026) than those with claudication.

### Perioperative complications

During the index admission, three (2.8%) early mortalities and two (1.9%) major amputations were recorded. All early mortalities and major amputations occurred in patients with CLTI.

In total, systemic/remote, local/vascular, and local/non-vascular complications occurred in 17 (16%), 11 (10%), and 11 (10%) patients, respectively (Supplementary Table 1). Among the systemic/remote complications, cardiac complications were the most common and occurred in 15 (14%) operations. However, the majority (73%) of the cases were classified as grade 1.

Local/vascular and systemic/remote complications were higher in patients with CLTI than those with claudication (16% vs. 4%, *P* = 0.042 for local/vascular complications; 23% vs. 8%, *P* = 0.033 for systemic/remote complications) (Supplementary Table 1).

### Patency rates and risk factors for PP loss

**Figure 2:**
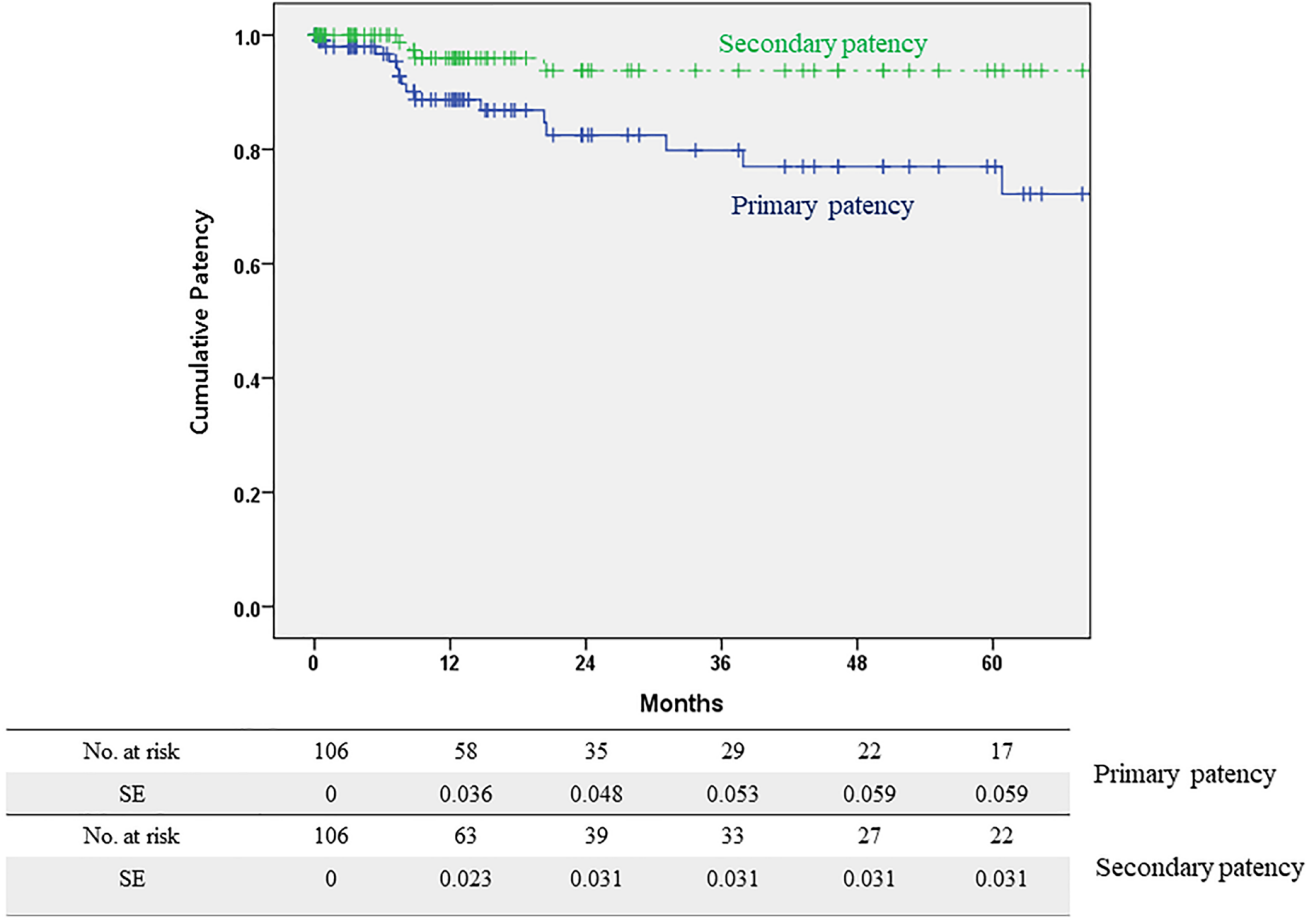
Primary and secondary patency rates of hybrid femoral endarterectomy with iliac angioplasty.

The median radiological follow-up duration was 14.5 months (interquartile range, 5.3–50.9). During the follow-up period, 17 PP loss events occurred. These events included additional iliac interventions due to restenosis in 11 limbs, FEIA occlusion in 5 limbs, and a patch infection requiring surgical intervention in 1 limb. The overall PP rates of FEIA were 88.7%, 79.8%, and 77.0% at 1, 3, and 5 years, respectively (Figure 2). Of the five FEIA occlusions, three limbs received a thrombectomy or thrombolysis, and the remaining two underwent a femorofemoral bypass. In addition, one patient with diffuse stenosis of FEIA and another with a patch infection received a femorofemoral bypass. In total, four FEIAs were abandoned during follow-up. The SP rates were 96.0% at 1 year and 93.8% at 3 and 5 years, respectively (Figure 2).

Table 4 outlines the risk factors for PP loss after the univariate analyses. Regarding the PP of FEIA, dialysis was associated with PP loss [hazard ratio (HR), 4.09; 95% confidence interval (CI), 1.13–14.75; *P* = 0.032] in the univariate analysis. Other risk factors, such as aortoiliac TASC II classification and iliac artery and CFA occlusion were not associated with PP loss after FEIA. Furthermore, the outflow status, such as femoropopliteal occlusion, did not affect patency.

**Table 4:** Risk factors for PP loss after univariate analysis.

	PP loss	
	Univariate analysis	
	HR (95% CI)	*P*-value
Sex, female	2.43 (0.68–8.76)	0.174
Age	0.99 (0.93–1.05)	0.677
CLTI	1.80 (0.66–4.92)	0.254
Previous ipsilateral revascularization	2.00 (0.63–6.35)	0.239
CIA and/or EIA occlusion	0.79 (0.22–2.81)	0.720
IIA occlusion	1.03 (0.38–2.77)	0.958
CFA occlusion	1.98 (0.73–5.38)	0.180
Femoropopliteal artery occlusion	0.94 (0.35–2.51)	0.898
DFA occlusion	1.40 (0.32–6.16)	0.660
Hypertension	1.63 (0.46–5.73)	0.449
Diabetes mellitus	0.45 (0.15–1.42)	0.173
Coronary artery disease	1.07 (0.39–2.96)	0.892
Congestive heart failure	1.18 (0.27–5.25)	0.824
Cerebrovascular disease	0.97 (0.34–2.80)	0.960
COPD	2.57 (0.70–9.37)	0.153
Renal insufficiency	1.26 (0.43–3.65)	0.672
Dialysis	4.09 (1.13–14.75)	0.032
Dyslipidemia	0.91 (0.33–2.46)	0.847
Infrainguinal revascularization	1.97 (0.69–5.74)	0.205
Profundaplasty	1.11 (0.35–3.48)	0.863
Iliac thrombectomy	0.88 (0.20–3.98)	0.871

HR, hazard ratio; CI, confidence interval; CLTI, chronic limb-threatening ischemia; CIA, common iliac artery; EIA, external iliac artery; IIA, internal iliac artery; CFA, common femoral artery; DFA, deep femoral artery; COPD, chronic obstructive lung disease.

## Discussion

This study presents the results of hybrid FEIA in AIOD involving the CFA. In our patients, all lesions were classified as TASC II C/D lesions, given that the involvement of the CFA in TASC II classification is considered a complex lesion. In addition, our patient cohort could be considered as having more complex type B lesions according to the GLASS because over 50% stenosis in the CFA was present in all patients. Despite this classification, FE with additional thrombectomy effectively induced classification shifts during the operation. In addition, the PP and SP demonstrated durable outcomes, with rates of 79.8% and 93.8% at 3 years and 77.0% and 93.8% at 5 years, respectively. Interestingly, the occlusion status of the iliac artery and CFA, usually related to poor patency after EVT, did not affect the patency after FEIA; therefore, hybrid FEIA emerges as a primary treatment option for AIOD with CFA involvement.

Traditionally, open surgical repair, such as aortofemoral or iliofemoral bypass, has been recommended for treating complex AIOD, ensuring long-term durability. However, the global increase in older adults with multiple comorbidities, precluding invasive surgery, has shifted the treatment landscape. In addition, given the excellent patency after CFA endarterectomy and the theoretical disadvantages of CFA stenting in high-mobility areas, hybrid FEIA has garnered global interest. Notably, the percentage of patients with peripheral arterial occlusive disease undergoing hybrid reconstruction in the US surged from 6.1% in 2010 to 32% in 2017 (9).

Remarkably, the preoperative TASC II classification did not impact PP in our series. According to the current TASC classification, the presence of CFA disease alongside any severity of iliac disease falls under TASC II C/D. However, in patients without long-segment iliac artery occlusion, treating CFA with FE will transform the classification of these lesions to TASC II A/B. The initial preoperative TASC classification in our series was C in 65 limbs and D in 41 limbs. Following FE, the majority of the AIOD cases were reclassified as simple lesions (TASC II A in 24 limbs and TASC II B in 69 limbs). In addition, only five limbs (4.7% of the total) retained a complex iliac lesion classification after additional thrombectomy. Therefore, FE and adjunctive thrombectomy can convert complex AIOD lesions into simple ones.

Table 5 lists the reported outcomes and patency after FE with iliac stent or stent graft placement. Although short- and medium-term patency after hybrid FEIA has been extensively reported (14–18, 21), there is a scarcity of studies on long-term patency after FEIA. Reported 5-year PP following FE with an iliac stent or stent graft ranges from 60% to 87% (19, 20, 22). In our patient cohort, the PP at 5 years after hybrid FEIA was 77.0%, aligning with previous research. Notably, the SP at 5 years reached 93.8%, indicating sustained functionality of most FEIAs during the follow-up period. The predominant cause of PP loss in our study was repeated EVT due to restenosis (65%, 11/17). Therefore, with a meticulous follow-up protocol after FEIA, achieving long-term durability seems feasible.

**Table 5:** Reported outcomes after femoral endarterectomy with iliac angioplasty.

	Years	Study type	*N*	Technical success	30-day mortality	1-year PP	3-year PP	5-year PP
Serna Santos et al. (14)	2023	Retro	163	88.3	8.1	96.6		
Starodubtsev et al. (15)	2022	Pros	102	98	0	93	91	
Bosse et al. (16)	2020	Retro	36		6.2	93.7		
Zavatta and Mell (17)	2018	Retro	1,472		1.8	79		
Ray et al. (18)	2018	Retro	41		2.7	85.4 (23 Mo)		
Maitrias et al. (19)	2017	Retro	127	100	0	91 (24 Mo)		87
Ilano et al. (20)	2017	Retro	111					73% for mild ID 68% for severe ID
Piazza et al. (21)	2011	Retro	84	99	1.1		91	
Chang et al. (22)	2008	Retro	193	98	2.3			60
Current study		Retro	106	100	2.8	88.7	79.8	77.0

PP, primary patency; Retro, retrospective; Pros, prospective; Mo, months; ID, iliac disease.

FE plays a central role in hybrid procedures for managing multilevel diseases involving the CFA. Isolated FE has been linked to excellent long-term patency, with a PP of over 90% at 3 years (8). However, wound complications and perioperative morbidities are its major disadvantages compared with EVT. Although FEIA has lower morbidity and mortality rates than the traditional open surgery for AIOD, it is not risk-free (17). Our study revealed that 16% of patients undergoing FEIA experienced systemic/remote complications, with cardiac complications being the most prevalent. In a study using the National Surgical Quality Improvement Program database in the US to evaluate perioperative complications following FE (23), pneumonia, cardiac arrest, myocardial infarction, and acute kidney injury accounted for 1.6%, 0.9%, 0.6%, and 0.3% of recorded complications, respectively. Notably, systemic/remote complications in our study surpassed those in the aforementioned study. However, iliac angioplasty was concurrently performed in all our patients, along with infrainguinal revascularization in a significant proportion. Furthermore, adhering to reporting guidelines, we included asymptomatic troponin-I elevation as a cardiac complication (12, 13). Indeed, four (3.8%) patients experienced fatal or symptomatic myocardial infarction in our study. In studies employing FEIA, myocardial ischemic events were reported in 4%–7% of participants (14, 22). Considering the more extensive nature of the surgery, including iliac angioplasty and infrainguinal revascularization, compared to FE alone, our results may be deemed acceptable. In addition, multivariate analysis of risk factors for systemic complications in our patients identified CLTI as an independent risk factor (adjusted HR, 3.45; 95% CI, 1.05–11.50, *P* = 0.041) (data not shown), emphasizing the need for vigilant monitoring of systemic complications in this population (24, 25).

This retrospective study has several limitations. First, the findings are derived from a single-center experience with a relatively modest number of patients, potentially limiting the generalizability of outcomes to broader populations with vascular diseases. Second, this study did not include a control group, such as patients who underwent open surgical repair or sole EVT for AIOD with CFA involvement. To determine the precise role of FEIA and the possibility of the primary revascularization method in the AIOD extending to the CFA, we need to evaluate and compare the outcomes of open surgical repair and EVT with those of the most advanced devices and techniques. However, according to our hospital's policy, in line with global trends and guidelines (9, 26), FEIA is preferred for these patients. Furthermore, this study included lesions with previous iliac intervention, and 12 limbs had this history. Their inclusion may result in selection bias because the treatment results may be affected by the previous iliac intervention. However, in a subgroup analysis of lesions with or without previous ipsilateral iliac intervention, the results showed no difference in PP (*P* = 0.981). Finally, a selection bias exists in determining hybrid FEIA in this series. During the study period, four vascular surgeons performed the operation. The operation methods may differ depending on the surgeons’ preference; hence, more severe forms of AIOD might have been treated with open surgery. Therefore, our series did not represent the overall cohort of patients with AIOD extending to the CFA; hence, the results should be interpreted cautiously.

## Conclusions

Despite the challenging nature of initially classified TASC II C/D lesions, our findings highlight the durable patency achieved with FEIA. FE demonstrated the potential to transform complex AIOD lesions into simpler ones, with no adverse impact on patency in cases of iliac artery and CFA occlusion after FEIA. Hybrid FEIA could be a viable primary treatment option, particularly for lesions featuring severe iliac and common femoral artery disease.

## Data Availability

The raw data supporting the conclusions of this article will be made available by the authors, without undue reservation.
