# Supplementary material for: Impact of open femoral endarterectomy on treating multilevel iliac and common femoral artery occlusive disease
Source: Front Surg. 2025 Jan 21;12:1445846. doi: 10.3389/fsurg.2025.1445846 (PMC11790649; doi:10.3389/fsurg.2025.1445846)
Supplement: Supplementary file 1 [file Table1.docx]

Supplementary Table 1. Early complications after hybrid femoral endarterectomy and iliac angioplasty with or without infrainguinal revascularization

|  | Total  (N = 106) | Claudication  (N = 50) | CLTI  (N = 56) | P value |
| --- | --- | --- | --- | --- |
| 30-day mortality | 3 (2.8%) | 0 | 3 (5%) | 0.245 |
| Major amputation | 2 (1.9%) | 0 | 2 (4%) | 0.497 |
| Local/Nonvascular | 11 (10.4%) | 3 (6%) | 8 (14%) | 0.163 |
| Hematoma | 5 | 2 | 3 |  |
| Grade 1 | 4 | 2 | 2 |  |
| Grade 2 | 1 | 0 | 1 |  |
| Lymphocele | 2 | 1 | 1 |  |
| Grade 1 | 1 | 1 | 0 |  |
| Grade 2 | 1 | 0 | 2 |  |
| Wound infection | 4 | 0 | 4 |  |
| Grade 1 | 1 | 0 | 1 |  |
| Grade 2 | 3 | 0 | 3 |  |
| Local/Vascular | 11 (10.4%) | 2 (4%) | 9 (16%) | 0.042 |
| Rupture | 4 | 1 | 3 |  |
| Grade 2 | 3 | 0 | 3 |  |
| Grade 3 | 1 | 1 | 0 |  |
| Macroembolism | 4 | 0 | 4 |  |
| Grade 1 | 1 | 0 | 1 |  |
| Grade 2 | 1 | 0 | 1 |  |
| Grade 3 | 2 | 0 | 2 |  |
| Thrombosis | 3 | 0 | 3 |  |
| Grade 3 | 3 | 0 | 3 |  |
| Dissection | 1 | 1 | 0 |  |
| Grade 2 | 1 | 1 | 0 |  |
| Systemic/Remote | 17 (16.0%) | 4 (8%) | 13 (23%) | 0.033 |
| Cardiac (Myocardial infarction) | 15 | 4 | 11 |  |
| Grade 1 | 11 | 4 | 7 |  |
| Grade 2 | 2 | 0 | 2 |  |
| Grade 3 | 2 | 0 | 2 |  |
| Respiratory | 4 | 0 | 4 |  |
| Grade 2 | 3 | 0 | 3 |  |
| Grade 3 | 1 | 0 | 1 |  |
| Renal | 2 | 1 | 1 |  |
| Grade 1 | 2 | 1 | 1 |  |
| Neurologic | 1 | 0 | 1 |  |
| Grade 2 | 0 | 0 | 1 |  |

CLTI; chronic limb-threatening ischemia
